# Adapting the Translational Science Benefits Model to improve health and advance health equity in diabetes: The Centers for Diabetes Translation Research Impact Framework

**DOI:** 10.1017/cts.2024.580

**Published:** 2024-09-23

**Authors:** Julie A. Schmittdiel, William H. Herman, Pamela Thornton, Marlon Pragnell, Debra Haire-Joshu

**Affiliations:** 1 Kaiser Permanente Northern California Division of Research, Pleasanton, CA, USA; 2 Department of Health System Sciences, Kaiser Permanente Bernard J Tyson School of Medicine, Pasadena, CA, USA; 3 University of Michigan Schools of Medicine and Public Health, Ann Arbor, MI, USA; 4 National Institute of Diabetes and Digestive and Kidney Diseases, National Institutes of Health, Bethesda, MD, USA; 5 American Diabetes Association, Arlington, VA, USA; 6 Washington University Brown School and School of Medicine, St. Louis, MO, USA

Translational research is broadly defined as research designed to take scientific discoveries “from the bench to the bedside and back again[1,2]” and seeks to “translate” research findings into improvements in human health [1]. While definitions of the different phases of translational research have evolved over time [1], the “T3” and “T4” phases of this research focus on disseminating and implementing the findings of efficacy research to result in broader effectiveness and improved health outcomes at the population level [1,3].

Despite the need for translational research to ultimately result in benefits to society [4], traditional metrics of research productivity are focused primarily on “bibliometrics” such as numbers of publications, citations, and successful grant applications [4]. The *Translational Science Benefits Model* (TSBM) was developed to provide benchmarks for assessing the real-world impact of translational research on enhanced public health and well-being [4]. The creators of the TSBM note that the uptake of this model for evaluating research impact “requires new partnerships between clinical scientists, academic administration, funders, and many relevant stakeholders [4].”

**Figure 1.**
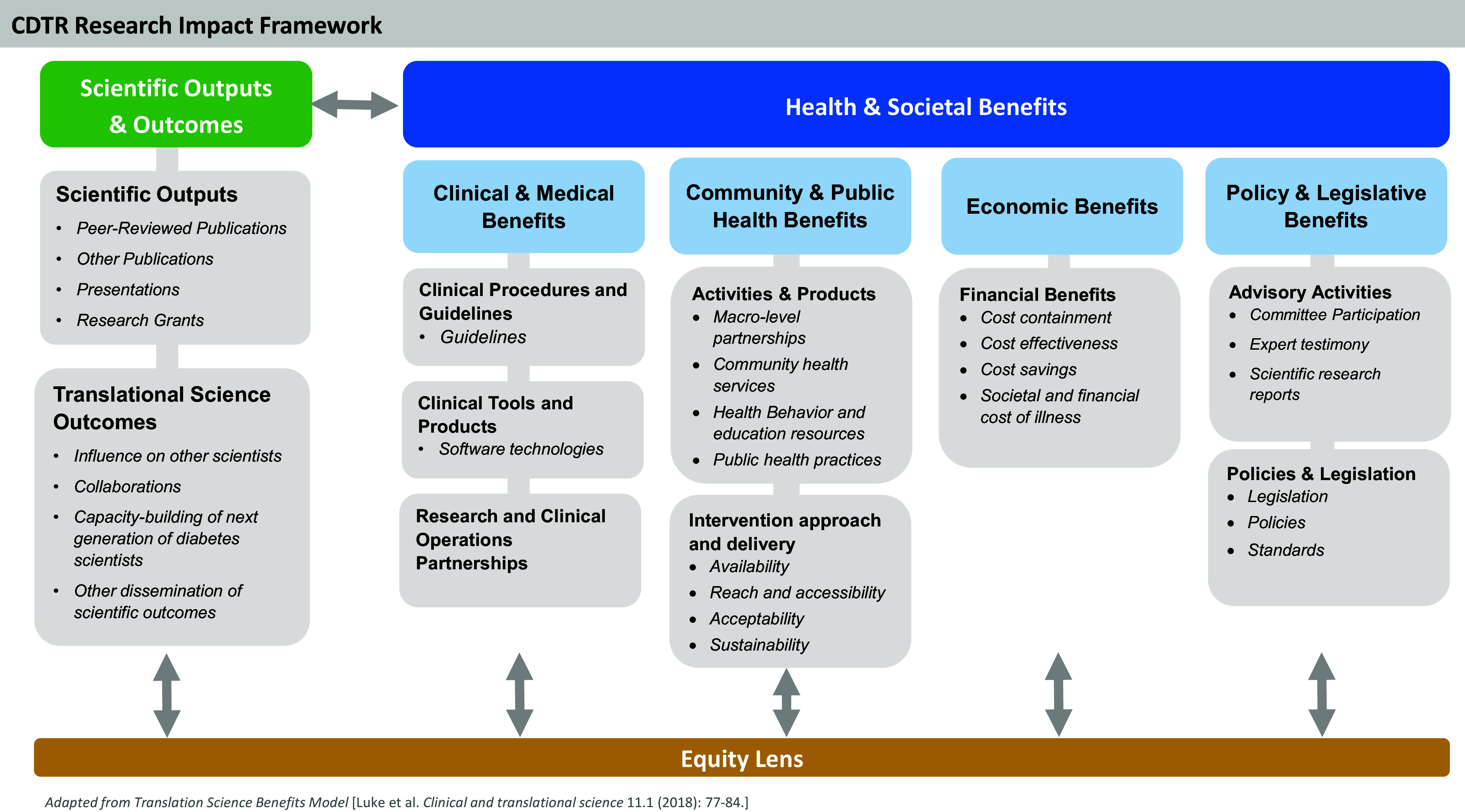
The Centers for Diabetes Translation Research (CDTR) Research Impact Framework.

The Centers for Diabetes Translation Research (CDTR) [5] leveraged a partnership between scientific investigators and program officials at the National Institute for Diabetes and Digestive and Kidney Diseases (NIDDK) to apply the TSBM framework to better understanding and measuring the impact of diabetes-focused translational research on health and health equity. The mission of the seven NIDDK-funded CDTRs is to translate the best evidence on diabetes care and prevention into practice to improve patient outcomes, address structural and societal barriers to health, and advance health equity [5]. The CDTRs have been very successful at achieving the traditional metrics of diabetes translation research, with over 4,800 publications and 580 extramural-funded grants credited to the CDTRs since the program’s inception in 2011. While NIH rightfully values these accomplishments as metrics of the CDTR program’s successes, the directors of the CDTRs and NIDDK sought to establish more comprehensive indicators of the CDTR’s productivity and impact by creating the **CDTR Research Impact Framework** (Figure 1).

The CDTR Research Impact Working Group, comprised of CDTR Directors, used an iterative process to adapt the TSBM domains and indicators to reflect the CDTR’s scope and mission. The CDTR Research Impact Framework (CDTR-RIF) leverages the existing structure of the TSBM to provide a framework for evaluating the comprehensive health and societal benefits of diabetes translational research to communities, clinicians, policymakers, and research funders. Like the TSBM, the CDTR-RIF focuses on assessing the “Health and Societal” benefits of research in four key domains: “Clinical and Medical” “Community and Public Health” “Economic” and “Policy and Legislative.” Drawing on the TSBM logic model [4], the CDTR-RIF also has a “Scientific Outputs and Outcomes” domain for translational research including both traditional metrics such as peer-reviewed publications and more translationally focused outcomes such as research’s influence on other scientists. The CDTR-RIF includes “Collaborations” in this domain to emphasize the need to create diverse, multisectorial partnerships to close the gap between research, practice, and real-world impact, and to provide robust platforms for conducting health equity research [5,6]. This domain also emphasizes the need for capacity-building to support the next generation of scientists working in diabetes translational research [7].

The CDTR-RIF’s “Health and Societal Benefits” of research focuses on benefits relevant to diabetes care and prevention, emphasizing the need to produce guidelines and health education resources and support diabetes-related health economic analyses, health policy, and legislation. For instance, CDTR investigators partnered with Parents as Teachers (PAT), a national home-visiting program promoting optimal child health and development through family support, to reduce the diabetes risk of parents from underrepresented groups by embedding a weight management curriculum within routine home-visiting practice [8,9]. The adapted curriculum was officially licensed and included within the required foundational training and practice of PAT home-visitors nationwide, yielding societal benefits by increasing equitable reach to all families. The CDTR-RIF enriches the TSBM by addressing “Health and Societal Benefits” such as “Research and Clinical Operations Partnerships” that are key to improving diabetes care outcomes [6], and the benefits derived through implementation of scientific advances to understand the “Availability,” “Reach and Accessibility,” “Acceptability,” and “Sustainability” of community-level interventions [10].

The CDTR-RIF underscores the critical need to view all potential translational research efforts through an “Equity Lens.” This emphasis on health equity as a guiding principle for diabetes translational research is consistent with the recommendations of the “NIDDK Pathways to Health for All” report released in May 2023 [11], and echoes its call for translational research to advance health equity, ameliorate health disparities, and embed the community in all research activities. It also is well-aligned with the U.S. Department of Health and Human Services “Healthy People 2030” plan to advance societal health by eliminating health disparities [12].

The CDTR-RIF serves as a guide to diabetes translation scientists in designing, implementing, and measuring the impact of research to benefit population and community health. This framework can also provide guidance to research funders seeking to maximize their funding’s impact. For example, the CDTR-RIF could be used to evaluate grantees’ annual progress reports and identify domains for future translational research funding. A critical role of the CDTRs is to provide pilot and feasibility (P&F) funding to investigators working in diabetes translational research. These P&F grants have funded over 145 early-stage investigators, providing “seed” funding for scientists new to diabetes translational research to collect feasibility data needed to successfully compete for larger extramural grants [6]. Recently, the American Diabetes Association (ADA) provided additional funding to expand the impact of the CDTR P&F program. Both NIDDK and the ADA could leverage the CDTR-RIF metrics to assess the success of the P&F program, including short- and long-term impacts of the P&F grants and their investigators on the field going forward.

The CDTR-RIF adapts the original TSBM specifically for diabetes research and creates a framework that researchers, funders, and policymakers can use to more completely assess the health and societal benefits of diabetes research grants and investments. This framework may significantly increase our understanding of the impact of diabetes care and prevention research on population health and health equity. Future research should examine the effect of the framework’s application on these outcomes.
